# Comprehensive Genomic Profiling for Precision Oncology: Analytical Validation and Clinical Utility in Solid Tumors

**DOI:** 10.3390/diagnostics16071087

**Published:** 2026-04-03

**Authors:** Ashis K. Mondal, Ashutosh Vashisht, Vishakha Vashisht, Nikhil S. Sahajpal, Nivin Omar, Sudha Ananth, Pankaj Kumar Ahluwalia, Jaspreet Farmaha, Jana Woodall, Ravindra Kolhe

**Affiliations:** 1 Department of Pathology, Medical College of Georgia, Augusta University, Augusta, GA 30912, USA; amondal@augusta.edu (A.K.M.); avashisht@augusta.edu (A.V.); vvashisht@augusta.edu (V.V.); naomar@emory.edu (N.O.); sananth@augusta.edu (S.A.); pahluwalia@augusta.edu (P.K.A.); jfarmaha@augusta.edu (J.F.); jawoodall@augusta.edu (J.W.); 2 Greenwood Genetic Center, Greenwood, SC 29646, USA; nsahajpal@ggc.org; 3 Department of Pathology and Laboratory Medicine, Emory University School of Medicine, Atlanta, GA 30307, USA

**Keywords:** comprehensive gene profiling, next-generation sequencing, solid tumors, variant detection, tumor mutational burden

## Abstract

**Background**: Comprehensive genomic profiling (CGP) is increasingly used in precision oncology to identify actionable genomic alterations and guide targeted therapies in solid tumors. However, the clinical implementation of CGP assays requires rigorous analytical validation to ensure accurate and reproducible detection of diverse genomic alterations across heterogeneous tumor samples. Despite rapid advancements in next-generation sequencing technologies, there remains a need for validated CGP platforms that demonstrate reliable performance and readiness for routine clinical use. **Methods**: This study evaluated the analytical and clinical performance of a CGP assay capable of detecting multiple genomic alteration types, including single nucleotide variants (SNVs), insertions/deletions (Indels), copy number variations (CNVs), gene fusions, and tumor mutational burden (TMB). Validation was conducted using patient-derived 117 FFPE tumor samples, external proficiency testing materials, and reference standards. Assay performance was assessed through comparison with orthogonal methods and through evaluation of reproducibility, limit of detection, and TMB concordance. **Results**: The assay demonstrated excellent analytical performance, achieving 100% sensitivity, specificity, and accuracy for variant detection across evaluated samples. Strong concordance was observed for TMB estimation (R^2^ = 0.9925), with consistent classification of TMB-high cases. The assay showed robust inter- and intra-run reproducibility and reliable detection of low-frequency variants. Limit-of-detection (LOD) analysis confirmed accurate SNV detection at approximately 1% variant allele frequency and reliable RNA fusion detection at low input levels. **Conclusions**: The validated CGP assay provides accurate, reproducible, and comprehensive detection of clinically relevant genomic alterations in solid tumors. These results support its suitability for routine clinical deployment, enabling reliable genomic profiling to inform precision oncology treatment decisions.

## 1. Introduction

The genomic landscape of cancer is characterized by a diverse tumor-specific molecular alterations that inform diagnostic, prognostic, and therapeutic strategies, thereby advancing precision oncology [1]. The advent of targeted therapies, driven by the identification of actionable genomic biomarkers, has transformed clinical management across multiple cancer types [2]. Regulatory bodies, including the U.S. Food and Drug Administration (FDA) and the European Medicines Agency (EMA), have accelerated approvals for genomically guided therapeutics, with a growing pipeline of clinical trials poised to further expand the repertoire of precision treatments [3,4]. In this context, next-generation sequencing (NGS) has emerged as a cornerstone technology for molecular profiling, enabling high-throughput, multiplexed detection of genetic variants across diverse tumor types [5,6]. While NGS offers unparalleled potential for comprehensive genomic analysis, its clinical utility is strongly influenced by the selection of gene panels. Small-scale DNA-based gene panels (typically 50–100 genes) are limited in scope, often failing to capture the full spectrum of clinically relevant alterations, including pan-tumor biomarkers such as microsatellite instability (MSI) and tumor mutational burden (TMB) [7,8]. These constraints underscore the need for more comprehensive genomic profiling approaches to fully realize the potential of personalized medicine.

In recent times, large-scale NGS panels, encompassing 500 or more genes, such as Tempus xT (648 genes) and MSK-IMPACT (468 genes), have become essential in clinical diagnostics [9,10]. These comprehensive genomic profiling (CGP) assays enable simultaneous detection of multiple variant classes, including single nucleotide variants (SNVs), insertions and deletions (indels), copy number variations (CNVs), gene fusions, and structural rearrangements, across a broad range of cancer-associated genes [11]. By identifying rare or low-frequency variants often missed by smaller panels, CGP provides a more complete molecular profile. This enables the identification of actionable alterations that guide targeted therapies and immunotherapies [12]. This strategy of concurrently assessing multiple biomarkers aligns with Current Procedural Terminology (CPT) requirements for detecting actionable markers that may support reimbursement. It also enhances clinical and economic value of large gene panels [13].

Despite the advantages of CGP, its implementation necessitates rigorous validation to ensure analytical reliability, particularly given the complexity of bioinformatic pipelines required for data interpretation [14]. Few commercial CGP assays, such as FoundationOne CDx (324 genes; Foundation Medicine) and Oncomine Comprehensive Assay v3 (501 genes; Thermo Fisher Scientific), have undergone extensive validation for diagnostic use [7,15,16]. This remains the exception rather than the rule. The continuous evolution of cancer genomics drives the development of novel panels to incorporate emerging oncogenic drivers, resistance mutations, and biomarkers identified through large-scale genomic studies and real-world evidence [17]; requires that additional panels undergo similar rigorous validation. Advances in sequencing chemistries, bioinformatics algorithms, and variant detection sensitivities further enable the design of more robust panels capable of detecting low-frequency variants with high reproducibility [18].

Beyond panel design, the post-sequencing analysis workflow poses significant challenges in clinical laboratories, where rapid turnaround times are critical for patient care. Current NGS workflows involve multiple labor-intensive steps. Raw FASTQ data are first demultiplexed and aligned to generate BAM files. These are then processed into variant call format (VCF) files and annotated, often using third-party tools, to produce clinically actionable reports [19]. This fragmented process demands specialized bioinformatics expertise, which may overwhelm resource-constrained laboratories. Moreover, existing solutions often fail to fully integrate variant annotation and prioritization within a clinically relevant context, relying on external platforms such as OncoKB or VarSome [20,21]. The absence of streamlined, end-to-end bioinformatics pipelines limits the scalability of CGP and exacerbates disparities in diagnostic capabilities, particularly for smaller laboratories.

To address these challenges and expand the repertoire of validated CGP assays, we present a comprehensive validation of the Illumina’s CGP, a 523-gene assay designed to detect SNVs, CNVs, indels, splice variants, and gene fusions across 55 fusions-associated genes, while simultaneously quantifying TMB and MSI [22] (Table S1). We evaluated its analytical performance using 112 patient samples and multiple reference materials, including Acrometrix Oncology Hotspot DNA Mutation Mix, Seraseq Lung and Brain CNV Mix, Seraseq RNA Gene Fusion v2, and CAP testing materials. Performance metrics included limit of detection (LOD), precision, and accuracy, and were compared with an established orthogonal CGP assay. We specifically analyzed the variant types required under the TA validation guidelines, in accordance with CPT standards, evaluating key performance characteristics including accuracy, precision, reportable range, analytical sensitivity, and analytical specificity. Given its robust performance and integrated bioinformatics capabilities, including the Illumina’s in-house DRAGEN platform for variant calling, we advocate for the adoption of Illumina’s CGP as a transformative assay for comprehensive cancer genomic profiling in precision oncology.

## 2. Materials and Methods

### 2.1. Samples

A cohort of 112 unique patient samples, comprising 48 females (median age 66) and 36 males (median age 63), encompassing 13 distinct tumor types, was curated from banked DNA/RNA extracted from formalin-fixed, paraffin-embedded (FFPE) specimens. The cohort was predominantly composed of colon (n = 27) and lung (n = 21) cancers, followed by brain (n = 14), ovarian (n = 12), and breast (n = 10) tumors, while the remaining tumor types were represented by smaller sample numbers (Table S2). Additionally, sixteen College of American Pathologists (CAP) proficiency testing samples (CAP-PT) with known single nucleotide variants (SNVs) were included. Reference DNA controls comprised the AcroMetrix Oncology Hotspot Control (#969056, Thermo Scientific, Fremont, CA, USA), harboring over 500 mutations across 53 genes from the Catalogue of Somatic Mutations in Cancer (COSMIC) database, including SNVs, multiple nucleotide variants, deletions, insertions, and complex variants of varying lengths, and the Seraseq Lung & Brain CNV Mix, +12 copies (#0710-0416, SeraCare Life Sciences, Milford, MA, USA). Analysis focused on 13 genes and 17 variants in the AcroMetrix control and three copy number variations (CNVs) in EGFR, MYCN, and MET for the Seraseq control. For RNA analysis, the Seraseq Fusion RNA Mix v2 (#0710-0127, SeraCare Life Sciences, Milford, MA, USA) served as the reference, containing 11 gene fusions, one exon-skipping variant, and one multi-exon deletion. All samples were previously sequenced using an orthogonal CGP assay, and variant concordance was evaluated. The study adhered to the Association for Molecular Pathology (AMP) and CAP guidelines for clinical assay validation, complied with the Declaration of Helsinki, and was approved by the Institutional Review Board (IRB #00000150, HAC IRB #611298) at Augusta University, GA, USA. Per IRB approval, all protected health information (PHI) was removed, and data were anonymized prior to analysis.

### 2.2. Nucleic Acid Extraction and Quantification

Prior to nucleic acid extraction, a minimum tumor cellularity threshold of 10% was established for macro-dissection. Tumor content in the samples was evaluated and annotated by a board-certified pathologist using hematoxylin and eosin-stained tissue sections. Macro-dissection was performed on two to five unstained 5 µm FFPE tissue sections, followed by deparaffinization and nucleic acid isolation. DNA and RNA were extracted using the QIAamp DNA FFPE Tissue Kit (#56404, QIAGEN, Germantown, MD, USA) and RNeasy FFPE Kit (#74404, QIAGEN, Germantown, MD, USA), respectively, per manufacturer protocols. Nucleic acid quality was assessed using a Nanodrop spectrophotometer (Thermo Fisher Scientfiic, Waltham, MA, USA), with an OD 260/280 ratio of 1.7–2.2 deemed acceptable. Double-stranded (ds) DNA concentration was quantified using the Qubit Broad Range DNA Kit (#Q33231, Thermo Fisher Scientfiic, Waltham, MA, USA), and RNA quality was evaluated with the HS RNA Qubit Kit (#Q32855, Thermo Fisher Scientfiic, Waltham, MA, USA). Samples meeting quality control criteria were diluted with RNase/DNase-free water to achieve the target nucleic acid concentration. The assay was optimized for a total dsDNA input of 120 ng and an RNA input of 64 ng for sequencing.

### 2.3. Preparation of DNA Cocktails

To evaluate the performance metrics, ten DNA cocktail mixtures were formulated by combining patient-derived DNA with CAP proficiency testing (CAP-PT) samples. Eight of these mixtures were composed of DNA from 52 unique patient samples, each harboring known SNVs or CNVs, blended with ten CAP-PT samples, each containing a single variant of interest previously validated in the laboratory. Additionally, two DNA cocktail mixtures were prepared, each incorporating six CAP-PT samples, with three CAP samples allocated per group.

### 2.4. Analytical Sensitivity (Limit of Detection; LOD)

To evaluate the LOD, as well as inter- and intra-run reproducibility of the assay, reference materials were sequenced across at least three independent runs, with each sample performed in triplicate. The LOD for SNVs was assessed using the AcroMetrix Oncology Hotspot Control (variant allele frequency [VAF] 5–15%), tested at full concentration (100%, 80 ng) and diluted to 62.5% (50 ng), 50% (40 ng), 25% (20 ng), 12.5% (10 ng), 10% (8 ng) and 1% (0.8 ng) with wild-type DNA to determine the minimum detectable VAF. The LOD for CNVs was evaluated using the SeraCare Lung and Brain CNV Mix, tested undiluted (100%, 12 copies) and diluted to 50% (6 copies) and 25% (3 copies) with wild-type DNA. For gene fusion analysis, the FGFR3-TACC3 fusion (391 copies/µL) served as the reference to calculate final fusion input. The Seraseq Fusion RNA Mix was tested at serial dilutions (100%, 75%, 50%, 25%, 5%, 1%, 0.5%). Wild-type DNA/RNA was supplemented across all dilutions to maintain a consistent 100% total input.

### 2.5. Library Preparation for Sequencing

Library preparation was performed according to the manufacturer’s protocol using the TruSight Oncology 500 High Throughput Library Preparation Kit (Illumina, San Diego, CA), employing a hybrid capture methodology. RNA samples were denatured and subjected to two-step cDNA synthesis, consisting of first-strand synthesis followed by second-strand synthesis. The resulting double-stranded cDNA was subsequently purified using a bead-based cleanup process, and prepared for downstream library construction. Genomic DNA (gDNA) and cDNA library preparation processes were conducted concurrently, adhering to an identical workflow. DNA fragmentation was performed using an ultrasonicator (Covaris, Woburn, MA, USA), yielding fragments of 90–250 bp with a target peak size of approximately 130 bp. Subsequent steps included end repair, A-tailing, and adapter ligation. Adapter-ligated cDNA and gDNA fragments were amplified via index PCR using UP-index primers. Target enrichment was performed via hybrid capture utilizing probes specific to 523 genes of interest for SNV/CNV and 55 gene fusions utilizing OPR1 probes for RNA and OPD2 probes for DNA, followed by capture, PCR-based amplification, purification, and quantification of dsDNA with the Qubit High Sensitivity Kit (#Q32854, Thermo Fisher Scientific, Waltham, MD, USA). Following library normalization, DNA (80%) and RNA (20%) libraries were pooled and prepared for sequencing.

### 2.6. Sequencing and Data Analysis

Sequencing was conducted using a 101 bp paired-end configuration with 8-base pair indexes across 218 cycles. Libraries were sequenced on the Illumina’s NextSeq 550 DX instrument with a V2 flow cell kit (Illumina, San Diego, CA, USA). Raw sequencing reads were converted to FASTQ format automatically in the Illumina BaseSpace hub and subsequently processed into variant calling format (VCF) files. These were analyzed for single nucleotide variants (SNVs) and insertions/deletions (indels) using the Variant Calling Assessment Tool (version 3.2.0, Illumina, San Diego, CA, USA). Variants with a variant allele frequency (VAF) of ≥1% to ≥5% and a total read depth ranging from 50× to >250× were filtered for inclusion. In addition, variant filtering was governed by platform-defined quality metrics, including read depth and base quality, as implemented within the BaseSpace analysis pipeline. Variants detected at lower allele frequencies were interpreted within the context of these quality parameters. The pipeline incorporates internal filtering mechanisms to reduce technical artifacts, including those associated with FFPE-derived samples. RNA data were analyzed using the Illumina’s TruSight Tumor (TST170) BaseSpace App (v2.0.2, Illumina, San Diego, CA, USA), with high-confidence variant data exported as .csv files. All detected alterations were reported in accordance with the joint consensus recommendations of the Association for Molecular Pathology (AMP), American Society of Clinical Oncology (ASCO), and College of American Pathologists (CAP).

## 3. Results

### 3.1. Sequencing Quality Metrics

Quality control (QC) metrics for DNA and RNA libraries were rigorously assessed to ensure robust sequencing performance (Table 1). For DNA libraries, the median insert size was 114.1 bp, with an average of 109.2 usable microsatellite instability (MSI) counts. Coverage metrics included a percentage of exon bases with coverage exceeding 50× (PCT_EXON_50×) of 99.1% and a percentage of target bases with coverage exceeding 250× (PCT_TARGET_250×) of 90.2%, as detailed in the accompanying table. For RNA libraries, the median insert size was 105.5 bp, with an average of 2.8 usable MSI counts. Coverage metrics showed PCT_EXON_50× of 9.2% and PCT_TARGET_250× of 2.9%. All recorded sequencing metrics conformed to the manufacturer’s recommended threshold values for validation samples.

These results confirm that all sequencing quality metrics met established validation thresholds, demonstrating robust library quality and reliable sequencing performance for both DNA and RNA libraries.

### 3.2. Comprehensive Detection and Validation of DNA Variants and RNA Fusions

**Figure 1:**
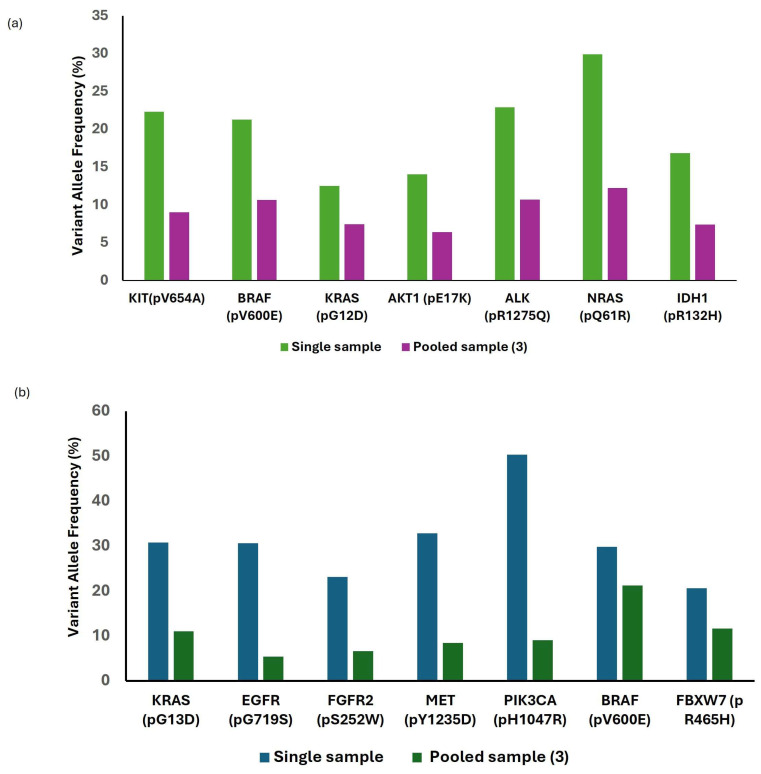
SNVs detected in undiluted and diluted (**a**) CAP-PT cocktail mix-1 and (**b**) CAP-PT cocktail mix-2.

Across 112 patient samples, 16 CAP proficiency testing (CAP-PT) samples, and all reference materials, a comprehensive analysis of 90 genes was conducted, identifying 117 variants, comprising 74 single nucleotide variants (SNVs), 4 copy number variations (CNVs), 3 insertions/deletions (indels), 1 duplication and 44 gene fusions (Tables S3–S6). All variants exhibited 100% concordance with orthogonal CGP assay results, demonstrating perfect sensitivity, specificity, and accuracy (Table 2). Furthermore, to evaluate the accuracy of CAP-PT cocktail, VAFs were compared between diluted and undiluted CAP-PT cocktail samples; although VAFs decreased with dilution, all variants remained detectable (Figure 1a,b). To assess the clinical accuracy of the assay for RNA, 27 patient samples representing diverse tumor types with known RNA fusions, which have been previously validated by orthogonal methods such as next-generation sequencing (NGS) or fluorescence in situ hybridization (FISH), were analyzed. All 33 distinct RNA fusion types were successfully detected in patient samples along with the 11 SeraSeq fusions.

Collectively, these findings demonstrate excellent analytical accuracy and concordance with orthogonal methods, confirming the assay’s capability to reliably detect diverse genomic alterations across multiple tumor types.

### 3.3. Assessment of LOD, and Inter and Intra-Run Analysis

**Figure 2:**
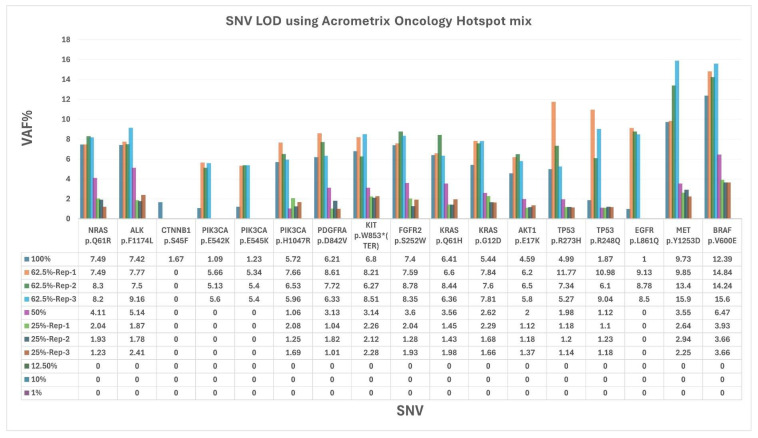
Evaluation of LOD of TO500 assay using Acrometrix Oncology Hotspot Mix.

The Acrometrix Oncology Hotspot DNA Mutation Mix was evaluated both undiluted (100%) and at serial dilutions of 62.5%, 50%, 25%, 12.5%, 10%, and 1% in an intra-run analysis to assess assay sensitivity. A progressive decline in sensitivity was observed with increasing dilution. At 100% DNA input (80 ng), all 17 SNVs across 13 genes were successfully detected (Figure 2). At 62.5% DNA input (50 ng), 16 SNVs were detected across triplicate analyses. At 50% (40 ng) and 25% (20 ng) DNA input levels, 13 SNVs were consistently identified. Notably, *CTNNB1 p.S45F* was only detected at 100% DNA input, while *PIK3CA p.E542K*, *PIK3CA p.E545K*, and *EGFR p.L861Q* remained detectable down to 62.5% DNA input. No SNVs were detected at 12.5% (10 ng), 10% (8 ng), or 1% (0.8 ng) DNA inputs. These findings suggest that a DNA input of 25% (20 ng) is sufficient for the detection of most SNVs under the assay conditions; however, consistent detection of all variants may require higher DNA input levels, indicating that analytical sensitivity varies depending on specific variant characteristics.

**Figure 3:**
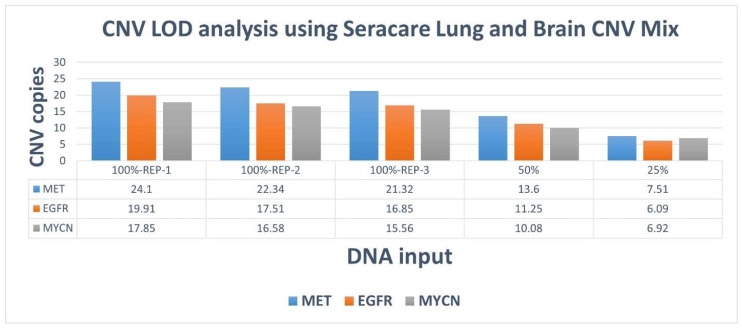
Evaluation of LOD of TO500 assay using Seraseq Lung and Brain CNV Mix.

The assay demonstrated a 100% accuracy for the Seraseq Lung and Brain CNV Mix, detecting all three CNVs (*EGFR*, *MET*, and *MYCN*) at both undiluted (100%, minimum 12 copies, run in triplicates as an intra run analysis) and diluted concentrations (50%, 6 copies and 25%, 3 copies) (Figure 3). Together, these results establish the assay’s sensitivity for detecting SNVs at low DNA input levels while maintaining high accuracy for CNV detection across varying copy number concentrations.

### 3.4. Inter Run Analysis

**Figure 4:**
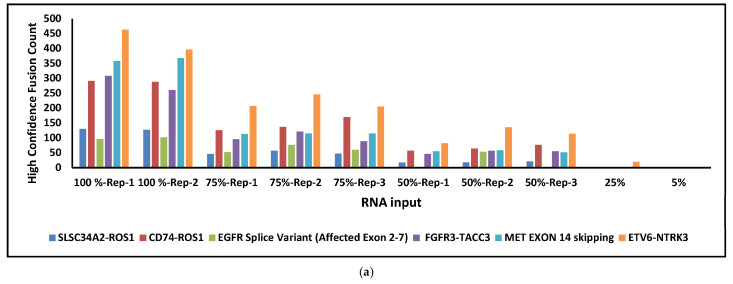

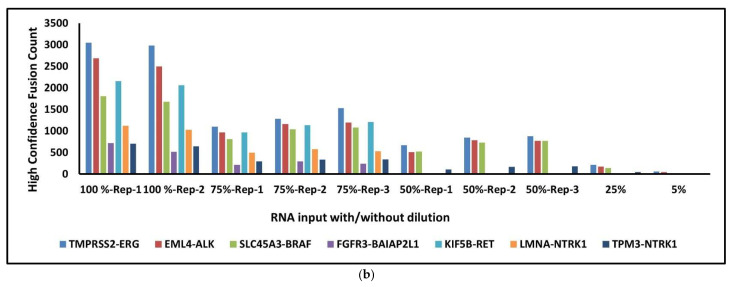
(**a**) Detection of RNA fusions and exonic variants, i.e., EGFR exons 2–7 splice variant and *MET* exon 14 skipping at varying input concentrations. (**b**) Other known RNA fusions.

To evaluate the limit of detection (LOD) for RNA fusions, the Seraseq Fusion RNA Mix, containing 11 fusion and exonic variants derived from two genes, was analyzed in an inter-run study. The Seraseq Fusion RNA Mix was serially diluted and analyzed at concentrations of 100% (in duplicate), 75% (in triplicate), 50% (in triplicate), 25%, 5%, 1%, and 0.5%. The panel of 11 known RNA fusions analyzed included *TMPRSS2-ERG*, *SLC34A2-ROS1*, *CD74-ROS1*, *EML4-ALK*, *SLC45A3-BRAF*, *FGFR3-BAIAP2L1*, *FGFR3-TACC3*, *NCOA4-RET*, *KIF5B-RET*, *TPM3-NTRK1*, *LMNA-NTRK1*, and *ETV6-NTRK3*. The *FGFR3-TACC3* fusion (391 copies/µL) served as the reference for calculating the final fusion input concentration. Additionally, exonic events such as the *EGFR* splice variant (exons 2–7) and *MET* exon 14 skipping were assessed (Figure 4a). At 100% and 75% input levels, all 13 RNA variants were successfully detected. At 50% input, 9 variants were identified, while 5 and 2 variants were detected at 25% and 5% input, respectively. No RNA fusions were detected at 1% and 0.5% input levels, attributed to insufficient supporting reads (Figure 4b).

**Figure 5:**
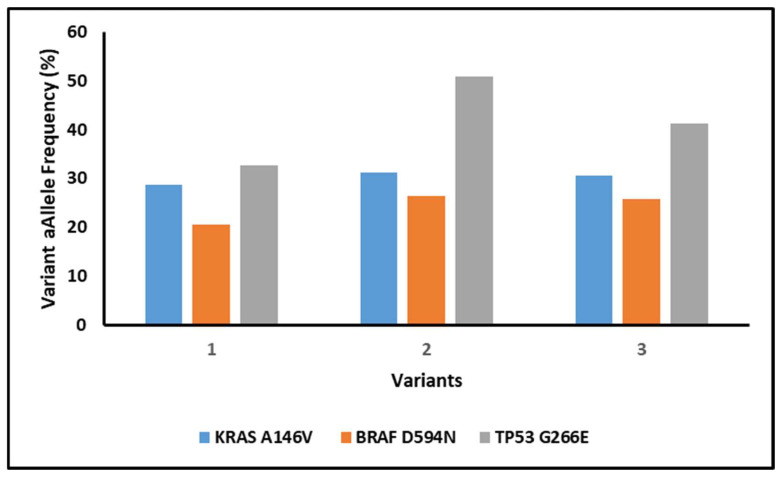
SNVs detected in patient samples in inter run analysis.

Additionally, the reproducibility and accuracy of the assay were assessed for three specific SNVs (*KRAS* p.A146V, *BRAF* p.D594N, *TP53* p.G266E) in patient samples. All three variants were successfully identified in inter-run analysis, demonstrating high degree of reproducibility and accuracy in detecting clinically relevant mutations (Figure 5).

These findings demonstrate the assay’s robust sensitivity for RNA fusion detection and high reproducibility across independent runs, supporting its reliability for identifying clinically relevant fusion events.

### 3.5. TMB Concordance Analysis Between TSO500 and FoundationOne Assays

**Figure 6:**
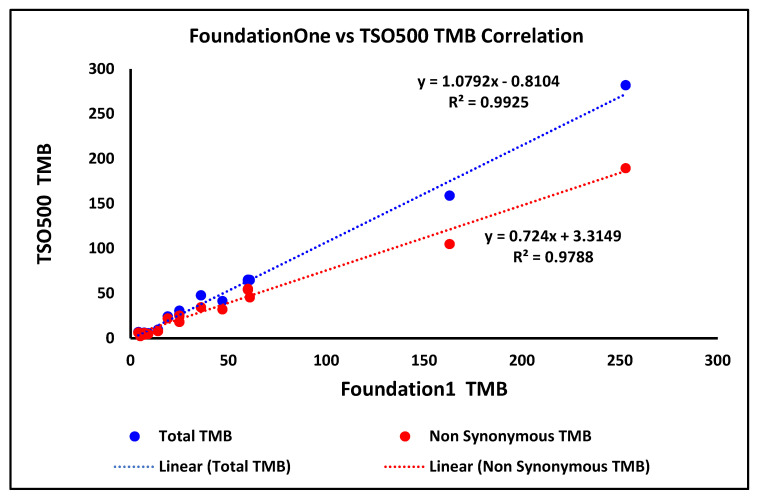
Pearson correlation of TMB score between the TSO500 and FoundationOne assay.

TMB concordance was assessed across a wide range of TMB values (5–253 mutations/megabase [mut/Mb]) by comparing results from the TSO500 assay to those obtained from 16 in-house patient samples previously tested by FoundationOne for both total and non-synonymous TMB scores. Using a TMB-high (TMB-H) threshold of ≥10 mut/Mb, 13 samples were classified as TMB-H by both assays, with 12 demonstrating concordant classification. One discordant sample exhibited a TMB of 14 mut/Mb with FoundationOne, whereas a TMB of 9 mut/Mb was observed with TSO500, resulting in a classification discrepancy. TMB concordance between TSO500 and FoundationOne is summarized in Figure 6. To quantitatively assess analytical concordance, Pearson correlation analysis was performed. The total and non-synonymous TMB scores demonstrated strong correlation, with R^2^ values of 0.9925 and 0.9788, respectively, supporting the high analytical agreement between the two assays.

Overall, the strong correlation and high classification concordance with FoundationOne support the analytical reliability of the assay for accurate TMB assessment in clinical samples.

## 4. Discussion

Here we present the validation of the Illumina’s CGP assay (TSO500) for comprehensive genomic profiling of solid tumor samples, demonstrating its robust performance across a broad spectrum of variant types, including SNVs, CNVs, indels, and RNA fusions. The results underscore the assay’s high sensitivity, specificity, and reproducibility, positioning it as a reliable tool for clinical next-generation sequencing (NGS) applications in precision oncology. These findings highlight the assay’s analytical and clinical validity, operational feasibility, and alignment with established benchmarks.

The TSO500 assay achieved 100% concordance with the orthogonal CGP assay across 112 patient samples, 16 CAP proficiency testing (CAP-PT) samples, and reference materials, detecting 117 variants with 100% sensitivity, specificity, and accuracy. Al-Kateb et al. (2025) reported similarly high precision (>93%) and accuracy (≥97%) for SNVs, indels, CNVs, and fusions in a pan-cancer cohort, validating TSO500’s performance with FFPE samples, consistent with our findings [23]. Kosco et al. (2021) further demonstrated TSO500’s reproducibility for CNV detection in FFPE samples, supporting our detection of *EGFR*, *MET*, and *MYCN* CNVs at low copy numbers (3 copies, 25% dilution) [24]. The assay’s ability to detect all expected variants, even at diluted variant allele frequencies (VAFs), underscores its robustness for low-frequency variants, which are critical in heterogeneous tumors. For the AcroMetrix Oncology Hotspot Control, target VAFs for genes *NRAS*, *ALK*, *CTNNB1*, *PIK3CA*, *PDGFRA*, *KIT*, *FGFR2*, *KRAS*, *AKT1*, and *TP53* ranged from 5% to 15%, while *EGFR, MET*, and *BRAF* had target VAFs of 15% to 35%, as specified by the supplier’s instructions. The assay’s ability to detect variants at a low DNA input of 25% (20 ng), corresponding to VAFs near 1%, underscores its sensitivity for low-frequency variants, which are critical in heterogeneous tumors. Zhao et al. (2020) reported 99% sensitivity and 100% specificity for variants at ≥5% VAF, aligning with our findings of consistent variant detection in diluted CAP-PT cocktail samples [25]. The validated performance of the TSO500 assay in this study supports its integration into routine clinical molecular diagnostics for comprehensive genomic profiling of solid tumors, suggesting that this platform has the potential to directly improve patient stratification and therapeutic decision-making in precision oncology.

The limit of detection (LOD) analysis confirmed the assay’s sensitivity for SNVs, with a minimum DNA input of 20 ng (25% dilution) sufficient to detect 13 of 17 variants, though certain variants (e.g., *CTNNB1* p.S45F) required higher inputs (80 ng). Kosco et al. (2022) reported 100% sensitivity for SNVs at 5% VAF with 40–60 ng DNA, suggesting that our observed LOD is within expected ranges for clinical reliability [24]. RNA fusions could be detected at as low as 5% input (Seraseq Fusion RNA Mix) highlighting TSO500’s capability to identify low-abundance fusion events, such as *FGFR3*-*TACC3* and *EML4*-*ALK*, which are actionable in NSCLC cancers [26,27]. Cuadras et al. 2023 demonstrated TSO500’s sensitivity for low-frequency variants (0.2–0.4% VAF) with 20 ng input in ctDNA, supporting our findings for RNA fusions at low inputs [28]. From a clinical implementation perspective, these findings have important implications for RNA input requirements in routine diagnostics. The progressive decline in fusion detection at lower input levels suggests that adequate RNA quantity and quality are critical for reliable fusion identification, particularly in FFPE-derived specimens where nucleic acid degradation and low tumor cellularity are common. In our study, consistent detection of all expected fusions was achieved at higher input levels (≥75%), while reduced detection at ≤50% input highlights the potential risk of false-negative results in samples with limited RNA yield. In real-world clinical settings, this is particularly relevant for small biopsies, cytology specimens, and samples with low tumor content, where RNA quantity may be suboptimal. Based on our findings, RNA input levels close to the recommended assay input (64 ng) are important for optimal fusion detection, as lower input levels were associated with a progressive decline in the number of detectable fusion events. Although selected fusions remained detectable at reduced input levels, comprehensive and consistent detection of all expected RNA variants was achieved only at higher input concentrations. These observations are consistent with prior studies demonstrating that RNA quality and input quantity significantly influence fusion detection performance in hybrid capture–based assays. Therefore, careful pre-analytical assessment of RNA quality and quantity remains a critical determinant of successful fusion detection in routine clinical practice.

Inter- and intra-run reproducibility, evidenced by consistent detection of SNVs (e.g., *KRAS* p.A146V, *BRAF* p.D594N, *TP53* p.G266E) and RNA fusions, adheres to the Association for Molecular Pathology (AMP) and College of American Pathologists (CAP) guidelines for clinical assay validation.

Furthermore, TMB concordance analysis revealed strong agreement between TSO500 and FoundationOne assays across 16 patient samples, with R^2^ values of 0.9925 (total TMB) and 0.9788 (non-synonymous TMB). Wei et al. (2022) reported a high correlation (R^2^ = 0.97) between TSO500 and FoundationOne TMB scores in 294 pan-tumor samples, consistent with our findings here [29]. Ramos et al. (2021) similarly noted a Pearson correlation of 0.93 in lung cancer, emphasizing TSO500’s reliability for TMB assessment [30]. Given TMB’s role as a predictive biomarker for immunotherapy response in cancers like non-small cell lung cancer (NSCLC) and melanoma [31,32], TSO500’s concordance supports its clinical utility for guiding immune checkpoint inhibitor therapy.

The SEQUOIA survey of nine laboratories revealed a median turnaround time of 14 days (range: 10–21 days) and an average monthly throughput of 8 samples, consistent with real-world data from Singh et al. (2020), who reported an average of 12–15 days turnaround times for comprehensive NGS panels [33]. The predominance of manual library preparation (8/9 laboratories) and variable hands-on times (2.5 h to 4 days) highlight opportunities for automation to enhance efficiency. The exclusive use of FFPE surgical and cytology samples aligns with TSO500’s design for FFPE-derived nucleic acids, as validated by Loderer et al. (2025), who reported high concordance for FFPE samples [34]. The use of NextSeq platforms along with Illumina’s in-house apps and third-party software such as PierianDx and Qiagen Cloud connect reflects standard clinical NGS practices, ensuring compatibility with existing infrastructure. Also, we specifically analyzed variants specified in the TA validation guidelines, as required by CPT standards, to ensure compliance with clinical reimbursement criteria. This adherence to regulatory standards enhances the assay’s applicability in clinical settings where reimbursement is contingent on validated performance metrics. Moreover, unlike several previously published studies that relied solely on patient samples or included limited sample sizes without extensive validation controls, our study integrates clinical specimens with standardized reference materials and proficiency testing samples. Furthermore, we systematically evaluated assay performance through inter- and intra-run reproducibility analyses while simultaneously assessing SNVs, CNVs, indels, RNA fusions, and TMB, thereby demonstrating the assay’s robustness and suitability for integrated genomic profiling in routine clinical practice.

Moreover, Illumina’s CGP assay concordance with orthogonal methods (e.g., FISH, NGS) for RNA fusions and TMB aligns with National Comprehensive Cancer Network (NCCN) guidelines for molecular testing. However, there are certain limitations that can be addressed in future studies. Validation studies like this need to include rare tumor types and Integrating machine learning-based bioinformatics, as suggested by Kaur et al. (2023), which could enhance variant calling accuracy for low-VAF variants [35]. Furthermore, the present study has uneven representation of tumor types, with several malignancies represented by a limited number of samples, which may restrict the generalizability of assay performance across all tumor entities.

Beyond these biological and cohort-related limitations, practical implementation of CGP assays in routine clinical laboratories introduces additional considerations related to workflow complexity and operational scalability. The overall workflow complexity associated with CGP assays, including multiple pre-analytical and analytical steps such as nucleic acid extraction, library preparation, hybrid capture, sequencing, and bioinformatics analysis, may introduce variability in routine clinical laboratory settings. Each of these steps is sensitive to sample quality, particularly for FFPE specimens, where DNA/RNA degradation and variable tumor purity can impact library yield and downstream performance. The manual nature of library preparation can further contribute to operator-dependent variability, affecting library quality, uniformity of coverage, and ultimately variant detection, especially in laboratories handling high sample volumes.

While our study demonstrated high reproducibility across repeated runs, maintaining consistent performance in routine practice requires stringent quality control measures, standardized operating procedures, and well-trained personnel. For example, variations in DNA input quantification or hybrid capture efficiency may influence sensitivity for low-frequency variants, particularly in samples with low tumor content. In addition, batching requirements—such as running 8–12 samples per sequencing run to optimize reagent utilization—may impact turnaround time in settings with fluctuating sample inflow, potentially delaying reporting in low-volume laboratories. Conversely, high-throughput centers may face challenges related to workflow coordination and data management due to increased sample load.

Scalability is also influenced by sequencing capacity, instrument availability, and bioinformatics infrastructure, including data storage and interpretation pipelines. Integration of automated library preparation systems has been shown to reduce hands-on time, minimize operator variability, and improve inter-run consistency. Similarly, implementation of standardized and automated bioinformatics workflows can enhance reproducibility and reduce interpretation variability. Collectively, these considerations highlight the importance of laboratory infrastructure, workflow optimization, and process standardization to ensure reliable and scalable implementation of comprehensive genomic profiling assays in routine clinical practice.

Although the assay demonstrated 100% sensitivity, specificity, and accuracy across all variant classes, certain variant categories, including CNVs, indels, and duplications, were represented by a limited number of events. Consequently, these performance estimates should be interpreted as descriptive measures, as small sample sizes may limit statistical precision and generalizability. Future studies should expand validation across additional rare tumor types and larger multi-institutional cohorts to further establish the assay’s clinical utility. Integration of automated library preparation workflows and advanced bioinformatics approaches, including machine learning–assisted variant interpretation, may further enhance sensitivity for low-frequency variants and improve scalability for high-throughput clinical testing.

## 5. Conclusions

The TSO500 assay demonstrates exceptional analytical and clinical validity for detecting SNVs, CNVs, indels, duplications, and RNA fusions in solid tumor samples, with high concordance to orthogonal methods and robust reproducibility. Its operational feasibility supports its integration into clinical NGS workflows, advancing precision oncology by enabling accurate identification of actionable alterations.

## Figures and Tables

**Table 1:** Sequencing Run QC metrics showing different parameters showing the DNA, RNA and respective standard deviation (SD) values.

Parameters	DNA	SD-DNA	RNA	SD-RNA
MEDIAN_INSERT_SIZE (bp)	114.1	7.43	105.54	12.65
PCT_EXON_50× (%)	99.1	0.25	9.22	1.10
USABLE_MSI_SITES (Count)	109.2	8.76	2.85	1.23
PCT_TARGET_250× (%)	90.2	5.02	2.86	0.61

**Table 2:** Diagnostic Precision and Concordance of Illumina’s CGP across Genomic Variant Classes.

Variant Type	Total Variants	Detected	Sensitivity (PPA) %	Specificity (NPA) %	Precision (PPV) %	NPV %	FNR %	FPR %	Accuracy %
SNVs	74	74	100	100	100	100	0	0	100
CNVs	4	4	100	100	100	100	0	0	100
INDELS	3	3	100	100	100	100	0	0	100
Duplications	1	1	100	100	100	100	0	0	100
Gene fusions	44	44	100	100	100	100	0	0	100

## Data Availability

All the relevant data is provided within the manuscript.

## Supplementary Materials

The following supporting information can be downloaded at: https://www.mdpi.com/article/10.3390/diagnostics16071087/s1, Table S1: Complete list of 523 genes and 55 gene fusions covered in the TSO500 Comprehensive genomic panel, Table S2: Showing the distribution of tumor types in the study, Table S3: Showing the total number of gene analyzed in the study, Table S4: Showing the SNVs analyzed in the study, Table S5: Showing the combined information of CNVs and Indels analyzed in the study, Table S6: showing the gene fusions studied.
