# Supplementary material for: Comprehensive Genomic Profiling for Precision Oncology: Analytical Validation and Clinical Utility in Solid Tumors
Source: Diagnostics (Basel). 2026 Apr 3;16(7):1087. doi: 10.3390/diagnostics16071087 (PMC13073334; doi:10.3390/diagnostics16071087)
Supplement: Supplementary file 1 [file diagnostics-16-01087-s001.zip › diagnostics-4142351-supplementary.pdf]

Table S1 Complete list of 523 genes and 55 gene fusions covered in the TSO500 Comprehensive genomic panel.

**TruSight Oncology 500**			
**Gene symbol**	**Small variants**	**Focal amplifications**	**Fusions (from RNA)**
ABL1	✓	-	✓
ABL2	✓	-	-
ACVR1	✓	-	-
ACVR1B	✓	-	-
AKT1	✓	-	-
AKT2	✓	✓	-
AKT3	✓	-	✓
ALK	✓	✓	✓
ALOX12B	✓	-	-
ANKRD11	✓	-	-
ANKRD26	✓	-	-
APC	✓	-	-
AR	✓	✓	✓
ARAF	✓	-	-
ARFRP1	✓	-	-
ARID1A	✓	-	-
ARID1B	✓	-	-
ARID2	✓	-	-
ARID5B	✓	-	-
ASXL1	✓	-	-
ASXL2	✓	-	-
ATM	✓	✓	-
ATR	✓	-	-
ATRX	✓	-	-
AURKA	✓	-	-
AURKB	✓	-	-
AXIN1	✓	-	-

AXIN2	✓	-	-
AXL	✓	-	✓
B2M	✓	-	-
BAP1	✓	-	-
BARD1	✓	-	-
BBC3	✓	-	-
BCL10	✓	-	-
BCL2	✓	-	✓
BCL2L1	✓	-	-
BCL2L11	✓	-	-
BCL2L2	✓	-	-
BCL6	✓	-	-
BCOR	✓	-	-
BCORL1	✓	-	-
BCR	✓	-	-
BIRC3	✓	-	-
BLM	✓	-	-
BMPR1A	✓	-	-
BRAF	✓	✓	✓
BRCA1	✓	✓	✓
BRCA2	✓	✓	✓
BRD4	✓	-	-
BRIP1	✓	-	-
BTG1	✓	-	-
BTK	✓	-	-
C11orf30	✓	-	-
CALR	✓	-	-
CARD11	✓	-	-
CASP8	✓	-	-
CBFB	✓	-	-
CBL	✓	-	-
CCND1	✓	✓	-
CCND2	✓	-	-

CCND3	✓	✓	-
CCNE1	✓	✓	-
CD274	✓	-	-
CD276	✓	-	-
CD74	✓	-	-
CD79A	✓	-	-
CD79B	✓	-	-
CDC73	✓	-	-
CDH1	✓	-	-
CDK12	✓	-	-
CDK4	✓	✓	✓
CDK6	✓	✓	-
CDK8	✓	-	-
CDKN1A	✓	-	-
CDKN1B	✓	-	-
CDKN2A	✓	-	-
CDKN2B	✓	-	-
CDKN2C	✓	-	-
CEBPA	✓	-	-
CENPA	✓	-	-
CHD2	✓	-	-
CHD4	✓	-	-
CHEK1	✓	✓	-
CHEK2	✓	✓	-
CIC	✓	-	-
CREBBP	✓	-	-
CRKL	✓	-	-
CRLF2	✓	-	-
CSF1R	✓	-	✓
CSF3R	✓	-	-
CSNK1A1	✓	-	-
CTCF	✓	-	-
CTLA4	✓	-	-

CTNNA1	✓	-	-
CTNNB1	✓	-	-
CUL3	✓	-	-
CUX1	✓	-	-
CXCR4	✓	-	-
CYLD	✓	-	-
DAXX	✓	-	-
DCUN1D1	✓	-	-
DDR2	✓	-	-
DDX41	✓	-	-
DHX15	✓	-	-
DICER1	✓	-	-
DIS3	✓	-	-
DNAJB1	✓	-	-
DNMT1	✓	-	-
DNMT3A	✓	-	-
DNMT3B	✓	-	-
DOT1L	✓	-	-
E2F3	✓	-	-
EED	✓	-	-
EGFL7	✓	-	-
EGFR	✓	✓	✓
EIF1AX	✓	-	-
EIF4A2	✓	-	-
EIF4E	✓	-	-
EML4	✓	-	✓
EP300	✓	-	-
EPCAM	✓	-	-
EPHA3	✓	-	-
EPHA5	✓	-	-
EPHA7	✓	-	-
EPHB1	✓	-	-
ERBB2	✓	✓	✓

ERBB3	✓	✓	-
ERBB4	✓	-	-
ERCC1	✓	✓	-
ERCC2	✓	✓	-
ERCC3	✓	-	-
ERCC4	✓	-	-
ERCC5	✓	-	-
ERG	✓	-	✓
ERRFI1	✓	-	-
ESR1	✓	✓	✓
ETS1	✓	-	✓
ETV1	✓	-	✓
ETV4	✓	-	✓
ETV5	✓	-	✓
ETV6	✓	-	-
EWSR1	✓	-	✓
EZH2	✓	-	-
FAM123B	✓	-	-
FAM175A	✓	-	-
FAM46C	✓	-	-
FANCA	✓	-	-
FANCC	✓	-	-
FANCD2	✓	-	-
FANCE	✓	-	-
FANCF	✓	-	-
FANCG	✓	-	-
FANCI	✓	-	-
FANCL	✓	-	-
FAS	✓	-	-
FAT1	✓	-	-
FBXW7	✓	-	-
FGF1	✓	✓	-
FGF10	✓	✓	-

FGF14	✓	✓	-
FGF19	✓	✓	-
FGF2	✓	✓	-
FGF23	✓	✓	-
FGF3	✓	✓	-
FGF4	✓	✓	-
FGF5	✓	✓	-
FGF6	✓	✓	-
FGF7	✓	✓	-
FGF8	✓	✓	-
FGF9	✓	✓	-
FGFR1	✓	✓	✓
FGFR2	✓	✓	✓
FGFR3	✓	✓	✓
FGFR4	✓	✓	✓
FH	✓	-	-
FLCN	✓	-	-
FLI1	✓	-	✓
FLT1	✓	-	✓
FLT3	✓	-	✓
FLT4	✓	-	-
FOXA1	✓	-	-
FOXL2	✓	-	-
FOXO1	✓	-	-
FOXP1	✓	-	-
FRS2	✓	-	-
FUBP1	✓	-	-
FYN	✓	-	-
GABRA6	✓	-	-
GATA1	✓	-	-
GATA2	✓	-	-
GATA3	✓	-	-
GATA4	✓	-	-

GATA6	✓	-	-
GEN1	✓	-	-
GID4	✓	-	-
GLI1	✓	-	-
GNA11	✓	-	-
GNA13	✓	-	-
GNAQ	✓	-	-
GNAS	✓	-	-
GPR124	✓	-	-
GPS2	✓	-	-
GREM1	✓	-	-
GRIN2A	✓	-	-
GRM3	✓	-	-
GSK3B	✓	-	-
H3F3A	✓	-	-
H3F3B	✓	-	-
H3F3C	✓	-	-
HGF	✓	-	-
HIST1H1C	✓	-	-
HIST1H2BD	✓	-	-
HIST1H3A	✓	-	-
HIST1H3B	✓	-	-
HIST1H3C	✓	-	-
HIST1H3D	✓	-	-
HIST1H3E	✓	-	-
HIST1H3F	✓	-	-
HIST1H3G	✓	-	-
HIST1H3H	✓	-	-
HIST1H3I	✓	-	-
HIST1H3J	✓	-	-
HIST2H3A	✓	-	-
HIST2H3C	✓	-	-
HIST2H3D	✓	-	-

HIST3H3	✓	-	-
HLA-A	✓	-	-
HLA-B	✓	-	-
HLA-C	✓	-	-
HNF1A	✓	-	-
HNRNPK	✓	-	-
HOXB13	✓	-	-
HRAS	✓	-	-
HSD3B1	✓	-	-
HSP90AA1	✓	-	-
ICOSLG	✓	-	-
ID3	✓	-	-
IDH1	✓	-	-
IDH2	✓	-	-
IFNGR1	✓	-	-
IGF1	✓	-	-
IGF1R	✓	-	-
IGF2	✓	-	-
IKBKE	✓	-	-
IKZF1	✓	-	-
IL10	✓	-	-
IL7R	✓	-	-
INHA	✓	-	-
INHBA	✓	-	-
INPP4A	✓	-	-
INPP4B	✓	-	-
INSR	✓	-	-
IRF2	✓	-	-
IRF4	✓	-	-
IRS1	✓	-	-
IRS2	✓	-	-
JAK1	✓	-	-
JAK2	✓	✓	✓

JAK3	✓	-	-
JUN	✓	-	-
KAT6A	✓	-	-
KDM5A	✓	-	-
KDM5C	✓	-	-
KDM6A	✓	-	-
KDR	✓	-	✓
KEAP1	✓	-	-
KEL	✓	-	-
KIF5B	✓	-	✓
KIT	✓	✓	✓
KLF4	✓	-	-
KLHL6	✓	-	-
KMT2B	✓	-	-
KMT2C	✓	-	-
KMT2D	✓	-	-
KRAS	✓	✓	-
LAMP1	✓	✓	-
LATS1	✓	-	-
LATS2	✓	-	-
LMO1	✓	-	-
LRP1B	✓	-	-
LYN	✓	-	-
LZTR1	✓	-	-
MAGI2	✓	-	-
MALT1	✓	-	-
MAP2K1	✓	-	-
MAP2K2	✓	-	-
MAP2K4	✓	-	-
MAP3K1	✓	-	-
MAP3K13	✓	-	-
MAP3K14	✓	-	-
MAP3K4	✓	-	-

MAPK1	✓	-	-
MAPK3	✓	-	-
MAX	✓	-	-
MCL1	✓	-	-
MDC1	✓	-	-
MDM2	✓	✓	-
MDM4	✓	✓	-
MED12	✓	-	-
MEF2B	✓	-	-
MEN1	✓	-	-
MET	✓	✓	✓
MGA	✓	-	-
MITF	✓	-	-
MLH1	✓	-	-
MLL	✓	-	✓
MLLT3	✓	-	✓
MPL	✓	-	-
MRE11A	✓	-	-
MSH2	✓	-	✓
MSH3	✓	-	-
MSH6	✓	-	-
MST1	✓	-	-
MST1R	✓	-	-
MTOR	✓	-	-
MUTYH	✓	-	-
MYB	✓	-	-
MYC	✓	✓	✓
MYCL1	✓	✓	-
MYCN	✓	✓	-
MYD88	✓	-	-
MYOD1	✓	-	-
NAB2	✓	-	-
NBN	✓	-	-

NCOA3	✓	-	-
NCOR1	✓	-	-
NEGR1	✓	-	-
NF1	✓	-	-
NF2	✓	-	-
NFE2L2	✓	-	-
NFKBIA	✓	-	-
NKX2-1	✓	-	-
NKX3-1	✓	-	-
NOTCH1	✓	-	✓
NOTCH2	✓	-	✓
NOTCH3	✓	-	✓
NOTCH4	✓	-	-
NPM1	✓	-	-
NRAS	✓	✓	-
NRG1	✓	✓	✓
NSD1	✓	-	-
NTRK1	✓	-	✓
NTRK2	✓	-	✓
NTRK3	✓	-	✓
NUP93	✓	-	-
NUTM1	✓	-	-
PAK1	✓	-	-
PAK3	✓	-	-
PAK7	✓	-	-
PALB2	✓	-	-
PARK2	✓	-	-
PARP1	✓	-	-
PAX3	✓	-	✓
PAX5	✓	-	-
PAX7	✓	-	✓
PAX8	✓	-	-
PBRM1	✓	-	-

PDCD1	✓	-	-
PDCD1LG2	✓	-	-
PDGFRA	✓	✓	✓
PDGFRB	✓	✓	✓
PDK1	✓	-	-
PDPK1	✓	-	-
PGR	✓	-	-
PHF6	✓	-	-
PHOX2B	✓	-	-
PIK3C2B	✓	-	-
PIK3C2G	✓	-	-
PIK3C3	✓	-	-
PIK3CA	✓	✓	✓
PIK3CB	✓	✓	-
PIK3CD	✓	-	-
PIK3CG	✓	-	-
PIK3R1	✓	-	-
PIK3R2	✓	-	-
PIK3R3	✓	-	-
PIM1	✓	-	-
PLCG2	✓	-	-
PLK2	✓	-	-
PMAIP1	✓	-	-
PMS1	✓	-	-
PMS2	✓	-	-
PNRC1	✓	-	-
POLD1	✓	-	-
POLE	✓	-	-
PPARG	✓	-	✓
PPM1D	✓	-	-
PPP2R1A	✓	-	-
PPP2R2A	✓	-	-
PPP6C	✓	-	-

PRDM1	✓	-	-
PREX2	✓	-	-
PRKAR1A	✓	-	-
PRKCI	✓	-	-
PRKDC	✓	-	-
PRSS8	✓	-	-
PTCH1	✓	-	-
PTEN	✓	✓	-
PTPN11	✓	-	-
PTPRD	✓	-	-
PTPRS	✓	-	-
PTPRT	✓	-	-
QKI	✓	-	-
RAB35	✓	-	-
RAC1	✓	-	-
RAD21	✓	-	-
RAD50	✓	-	-
RAD51	✓	-	-
RAD51B	✓	-	-
RAD51C	✓	-	-
RAD51D	✓	-	-
RAD52	✓	-	-
RAD54L	✓	-	-
RAF1	✓	✓	✓
RANBP2	✓	-	-
RARA	✓	-	-
RASA1	✓	-	-
RB1	✓	-	-
RBM10	✓	-	-
RECQL4	✓	-	-
REL	✓	-	-
RET	✓	✓	✓
RFWD2	✓	-	-

RHEB	✓	-	-
RHOA	✓	-	-
RICTOR	✓	✓	-
RIT1	✓	-	-
RNF43	✓	-	-
ROS1	✓	-	✓
RPS6KA4	✓	-	-
RPS6KB1	✓	✓	✓
RPS6KB2	✓	-	-
RPTOR	✓	-	-
RUNX1	✓	-	-
RUNX1T1	✓	-	-
RYBP	✓	-	-
SDHA	✓	-	-
SDHAF2	✓	-	-
SDHB	✓	-	-
SDHC	✓	-	-
SDHD	✓	-	-
SETBP1	✓	-	-
SETD2	✓	-	-
SF3B1	✓	-	-
SH2B3	✓	-	-
SH2D1A	✓	-	-
SHQ1	✓	-	-
SLIT2	✓	-	-
SLX4	✓	-	-
SMAD2	✓	-	-
SMAD3	✓	-	-
SMAD4	✓	-	-
SMARCA4	✓	-	-
SMARCB1	✓	-	-
SMARCD1	✓	-	-
SMC1A	✓	-	-

SMC3	✓	-	-
SMO	✓	-	-
SNCAIP	✓	-	-
SOCS1	✓	-	-
SOX10	✓	-	-
SOX17	✓	-	-
SOX2	✓	-	-
SOX9	✓	-	-
SPEN	✓	-	-
SPOP	✓	-	-
SPTA1	✓	-	-
SRC	✓	-	-
SRSF2	✓	-	-
STAG1	✓	-	-
STAG2	✓	-	-
STAT3	✓	-	-
STAT4	✓	-	-
STAT5A	✓	-	-
STAT5B	✓	-	-
STK11	✓	-	-
STK40	✓	-	-
SUFU	✓	-	-
SUZ12	✓	-	-
SYK	✓	-	-
TAF1	✓	-	-
TBX3	✓	-	-
TCEB1	✓	-	-
TCF3	✓	-	-
TCF7L2	✓	-	-
TERC	✓	-	-
TERT	✓	-	-
TET1	✓	-	-
TET2	✓	-	-

TFE3	✓	-	-
TFRC	✓	✓	-
TGFBR1	✓	-	-
TGFBR2	✓	-	-
TMEM127	✓	-	-
TMPRSS2	✓	-	✓
TNFAIP3	✓	-	-
TNFRSF14	✓	-	-
TOP1	✓	-	-
TOP2A	✓	-	-
TP53	✓	-	-
TP63	✓	-	-
TRAF2	✓	-	-
TRAF7	✓	-	-
TSC1	✓	-	-
TSC2	✓	-	-
TSHR	✓	-	-
U2AF1	✓	-	-
VEGFA	✓	-	-
VHL	✓	-	-
VTCN1	✓	-	-
WISP3	✓	-	-
WT1	✓	-	-
XIAP	✓	-	-
XPO1	✓	-	-
XRCC2	✓	-	-
YAP1	✓	-	-
YES1	✓	-	-
ZBTB2	✓	-	-
ZBTB7A	✓	-	-
ZFHX3	✓	-	-
ZNF217	✓	-	-
ZNF703	✓	-	-

ZRSR2	✓	-	-

Table S2 Showing the distribution of tumor types in the study

**S.No.**	**Tumor Type**	**No. of samples**
1	Bladder	1
2	Brain	14
3	Breast	10
4	Cancer of Unknown Primary	1
5	Colon	27
6	Gastrointestinal Stromal Tumors (GIST)	3
7	Kidney	1
8	Liver	1
9	Lung	21
10	Melanoma	2
11	Neuroendocrine tumors	1
12	Ovarian	12
13	Prostatic Adenocarcinoma	1

Table S3: showing the total number of gene analyzed in the study

S.No.	**Genes**
1	AKT1
2	ALK

3	APC
4	AR
5	ATF1
6	ATM
7	BAIAP2L1
8	BARD1
9	BRAF
10	BRCA1
11	BRCA2
12	BRIP1
13	CASC3
14	CASZ1
15	CCAG8
16	CCDC170
17	CD74
18	CDK12
19	CHEK1
20	CHEK2
21	CLIP2
22	CTNNA2
23	CTNNB1
24	DEC1
25	DNAH12
26	EGFR
27	EML4
28	ERG
29	ESR1
30	ETV1
31	ETV6
32	EWSR1
33	FANCA

34	FBXW7
35	FGFR2
36	FGFR3
37	FIP1L1
38	FLT1
39	GTF2I
40	IDH1
41	IDH2
42	JDP2
43	KCNIP1
44	KDM4C
45	KIAA1549
46	KIF5B
47	KIT
48	KMT2A
49	KRAS
50	LMNA
51	MET
52	MLLT3
53	MSH2
54	MSH6
55	MTHFD1L
56	NEK9
57	NR4A3
58	NRAS
59	NRG1
60	NTRK1
61	NTRK3
62	PAX3
63	PDGFRA
64	PIK3CA

65	PLXDC1
66	PMS2
67	PPAT
68	PRKAA2
69	PRSS23
70	RAB3IP
71	RAD51B
72	RAD51D
73	RAD54L
74	RBFOX
75	RET
76	ROS1
77	RPS6KB1
78	SEPT14
79	SLC5A12
80	SLSC34A2
81	SLC45A3
82	TACC3
83	TMPRSS2
84	TP53
85	TPM3
86	VHL
87	VMP1
88	ZC2HC1B
89	ZNF507
90	ZNF512

Table S4: showing the Single nucleotide Variants (SNVs) analyzed in the study

S.No.	**SNVs**
1	AKT1 p.E17K
2	ALK R1275Q
3	ALK R1275Q
4	APC p.H1965R
5	AR p.E155K
6	AR p.V132I
7	ATM p.A112T
8	ATM p.C107Y
9	ATM p.T2438I
10	BARD1 (p.M414L)
11	BRAF V600E
12	BRCA1 p.E23fs
13	BRCA1 p.Q491*
14	BRCA2 p.V1862fs
15	BRIP1 p.W468R
16	CDK12 p.Q1291fs
17	CHEK1 p.K166N
18	CHEK2 p.R318C
19	CTNNB1 p.D32N
20	CTNNB1 p.S37C
21	CTNNB1 p.S45F
22	CTNNB1 p.T41A
23	EGFR G719X
24	EGFR L858R
25	EGFR L861Q
26	EGFR S768I

27	EGFR T790M
28	FANCA p.R29S
29	FBXW7 R465H
30	FGFR2 p.S252W
31	FGFR2 S252W
32	FGFR3 p.R248C
33	FGFR3 p.S249C
34	FGFR3 p.Y373C
35	IDH1 R132H
36	IDH2 p.R140Q
37	KIT p.P468Q
38	KIT p.P468Q
39	KIT p.W853*(TER)
40	KIT V654A
41	KRAS G12A
42	KRAS G12C
43	KRAS G12D
44	KRAS G12V
45	KRAS G13D
46	KRAS p.A146T
47	KRAS p.Q61H
48	MET p.Y1253D
49	MSH2 (p.A256T)
50	MSH6 p.P831L
51	NRAS p.Q61R
52	NRASp.Q61K
53	PDGFRA p.A820S
54	PDGFRA p.D842V
55	PDGFRA p.E263*
56	PDGFRA p.I657T
57	PDGFRA p.L593P

58	PDGFRA p.P1021T
59	PDGFRA p.R558C
60	PIK3CA p.E542K
61	PIK3CA p.H1047R
62	PIK3CA p.p.E545K
63	PMS2 p.V380G
64	RAD51B p.R159C
65	RAD51D p.T27I
66	RAD54L p.S636T
67	TP53 p.H178Sfs*69
68	TP53 p.H179R
69	TP53 p.M237I
70	TP53 p.Q167Afs*13
71	TP53 p.R248Q
72	TP53 p.R273H
73	TP53 p.Y234C
74	VHL p.L188Q

Table S5: showing the combined information of CNVs and Indels analyzed in the study

**S.NO.**	**CNVs**	**Indels**
1	EGFR	EGFR Exon 19-DEL & exon 20
2	ERBB2	ERBB2 p.G776delinsVV
3	MET	KIT p.D579del
4	MYCN	

Table S6: showing the gene fusions studied

S.No.	**Fusions**
1	GTF2I – BRAF
2	CLIP2 – BRAF
3	JDP2 – AR
4	PPAT-PDGFRA
5	PPAT – FIP1L1
6	EWSR1 – FLT1
7	ESR1-MTHFD1L
8	ESR1 – RAB3IP
9	ESR1 – ZC2HC1B
10	EWSR1 – ATF1
11	CASC3 – RPS6KB1
12	RPS6KB1 – VMP1
13	ERBB2 – PLXDC1
14	PRKAA2 – NOTCH2
15	EWSR1 – NR4A3
16	PRSS23 – KMT2A
17	ESR1 – CCDC170
18	CASZ1 – NOTCH2
19	EML4 – ALK
20	TPM3 – NTRK1
21	ZNF512 – ALK
22	DEC1 – ETV1
23	CTNNA2 – PAX3
24	DNAH12 – MET
25	NOTCH2 – NEK9
26	RBFOX – EML4

27	SLC5A12 – NOTCH2
28	ZNF507 – MET
29	NRG1 – KCNIP1
30	BRAF – KIAA1549
31	CCAG8 – AKT3
32	SEPT14 – EGFR
33	KDM4C – MLLT3
34	SLSC34A2-ROS1
35	CD74-ROS1
36	EGFR Splice Variant (Affected Exon 2-7)
37	FGFR3-TACC3
38	MET EXON 14 skipping
39	ETV6-NTRK3
40	TMPRSS2-ERG
41	SLC45A3-BRAF
42	FGFR3-BAIAP2L1
43	KIF5B-RET
44	LMNA-NTRK1
